# Analysis of the potential inappropriate use of medications in pediatric outpatients in China

**DOI:** 10.1186/s12913-021-07300-8

**Published:** 2021-11-25

**Authors:** Jing Cui, Lei Zhao, Xianghong Liu, Mengyujie Liu, Lihong Zhong

**Affiliations:** Department of Pharmacy, Qilu Hospital of Shandong University, Jinan, China

**Keywords:** Prescription, Pediatric, Outpatient, Drug utilization, Inappropriate medicine use

## Abstract

**Background:**

The appropriate use of medications is essential in children. Yet, detailed information on how drugs are being prescribed and dispensed to pediatric populations is not documented in China.

**Aim:**

The study objective was to analyze the details of medicine use and categorize the types of inappropriate use of medications on children.

**Methods:**

A retrospective cross-sectional study was conducted on the prescriptions of pediatric outpatients aged < 18 years from 2019 to 2020 at a major Chinese tertiary academic center. Each age group’s demographic and clinical characteristics were collected, and the ratios of inappropriate prescriptions were analyzed.

**Results:**

The total number of pediatric outpatients was 652,152, and 49.37% (322000) were prescribed medications, in which the most widely used medicines were respiratory, anti-infectives, and Traditional Chinese Medicines (TCMs). The prevalence rate of inappropriate prescriptions reached 20.49%, and in 2019 it was higher (21.71%) than that in 2020 (18.36%). The top three common inappropriate categories were indication-related off-label drug use, improper administration frequency, and overdosing, accounting for 67.93, 17.80 and 11.06% of all inappropriate prescriptions, respectively. The inappropriate prescriptions were more likely seen in patients aged 2–5 years and respiratory medicines.

**Conclusions:**

The study findings indicate that inappropriate drug use in pediatric outpatients is still common, and great attention needs to be paid. More prospective trials are required to identify the effectiveness, safety, and necessity of off-label drug use of medicines in children.

## Introduction

Data from the sixth national census shows that the population in the age range of 0–14 years reached 253 million, accounting for 17.95% of the total population in 2020 in China [1]. The number of children who get sick each year accounts for about 20% of the total number of patients [2]. The children’s unique physiological characteristics result in significantly different absorption, distribution, metabolism, and excretion of drugs compared to adults [3]. The potential of injury is higher in young children and infants. It can result in severe morbidity and even mortality, so the choice and use of drugs in children should be paid more attention [4]. However, unlicensed and off-label prescriptions for children are common in many countries [5–7]. Monitoring the safety of medicine use in children is very important, especially the use of medicines outside the specifications described in the license (e.g., in terms of formulation, indications, contraindications, or age) constitutes off-label and off-license use, and these are a significant area of concern [8].

In current studies, the information on medicine use was mostly collected from the national pharmaceutical claims database or nationwide health and Welfare’s registries, but these databases did not provide the rationality of drug use [9–11]. The details of how drugs are being prescribed and dispensed to pediatric populations are not available for the Chinese population. Understanding this information can identify areas of process improvement and can enhance the patient safety profile.

## Method

A retrospective cross-sectional study was conducted on a major Chinese tertiary academic center to analyze the details of medicine use and categorize the types of inappropriate use of medications in children. Ethical approval was granted by the Medical Ethics Committee of Qilu Hospital of Shandong University (Approval number KYLL-202011–136).

### Data sources

The prescriptions of pediatric outpatients aged < 18 years prescribed during January 1, 2019 and December 31, 2020 were extracted from the Hospital Information System (HIS) and Clinical rational drug use monitoring system. If the patient did not purchase the medicine prescribed by the doctor from the hospital, the medication records were missing in HIS, thus, this part of the data was excluded.

Patients were grouped into one of four age groups (< 2, 2–5, 6–11, and 12–17 years) [9, 12–15], and the following information on patient’s prescription was collected: age, sex, insurance type, medication name, and dosage, administration route and spending of drugs (converted from Chinese Yuan to US$ based on the exchange rate of 6.90 Yuan per US$,which was the average exchange rate for 2019 and 2020). Medications were classified based on the Anatomical Therapeutic Chemical (ATC 2021) classification proposed by the World Health Organization (WHO) [16]. The medications not registered in ATC were classified based on their clinical applications.

### Inappropriate prescription categories

The rationality of prescriptions was analyzed by the Clinical rational drug use monitoring system, which was developed by the author’s hospital to monitor the inappropriate use of drugs. The definition and classification of inappropriate prescriptions in this system refer to the Hospital Prescription Review Management Specification (Trial) issued by the National Health Commission of the People’s Republic of China, and it can automatically review, analyze and summarize the rationality of prescription drug use [13].

Inappropriate prescriptions were categorized into the following nine categories: (1) overdosing, (2) improper administration frequency, (3) improper administration routes, (4) contraindication, (5) improper diluent volume, (6) medications not allowed for pediatric patients, (7) improper drug combination, (8) repeated administration, and (9) indication-related off-label drug use.

### Statistical analysis

All analyses were conducted using SPSS Statistics 25.0 (IBM, Armonk, N Y). A descriptive analysis was conducted on the patient’s demographic, clinical, and medication characteristics. Mean (SD) was computed for continuous data. Frequencies and percentages were calculated for categorical variables. The prevalence and inappropriate medicine use changes were also analyzed for all the children between 2019 and 2020. The Chi-square test was used to compare the differences in the prevalence rates, and the T-test was used to determine the significance of differences between means.

## Results

The total number of pediatric outpatients was 652,152, of which 59.09% were boys, and 49.37% (322000) were prescribed medications. Among patients who received medications, 27.04% received injected formulations, and basic medical insurance covered 1.13%. The total spending on drugs was US$9.86 million, an average of 2.40 kinds of medications and US$30.61 per patient per visit. The prescription details of demographic and drug character were showed in Table 1.

**Table 1:** Prescription status by age groups and years

	Age group (years)								All children	
	< 2		2–5		6–11		12–17			
	2019	2020	2019	2020	2019	2020	2019	2020	2019	2020
Number of pediatric outpatients	55,936	36,841	126,443	76,646	116,697	88,510	83,498	67,581	382,574	269,578
Boys (%)	59.41%	57.72%	58.82%	60.28%	59.93%	59.46%	59.71%	56.03%	59.44%	58.59%
Number of pediatric prescriptions	33,088	15,071	72,542	36,566	60,355	35,533	38,621	30,224	204,606	117,394
Proportion of children received medications	59.15%	40.91%	57.37%	47.71%	51.72%	40.15%	46.25%	44.72%	53.48%	43.55%
Average number of medicines prescribed for each child	2.47	2.12	2.57	2.27	2.32	1.98	2.63	2.62	2.49	2.25
Average spending on drugs of one child in each visit (dollars)	17.27	16.07	18.98	20.46	31.79	43.40	48.43	52.52	28.04	35.09
Number of children covered by basic medical insurance	155	37	803	352	653	547	587	494	2198	1430
Proportion of children received injected medications	34.38%	23.53%	34.07%	27.24%	26.66%	21.58%	21.04%	18.49%	29.48%	22.80%
Proportion of injections in all the medicines used	54.18%	41.37%	54.70%	42.64%	44.71%	31.11%	36.82%	34.02%	48.31%	36.84%

The proportion of children who received medications (RR 0.814; 95%CI 0.810–0.819; *p* < 0.001), the average number of medications prescribed (*p* < 0.001), and the proportion of children who received injected formulations (RR 0.773; 95%CI 0.765–0.783; *p* < 0.001) decreased significantly in 2020 compared with 2019. The spending of drugs on one child in each visit increased with age and was higher in 2020 than that in 2019 (*p* < 0.001).

### Drug utilization

**Fig. 1:**
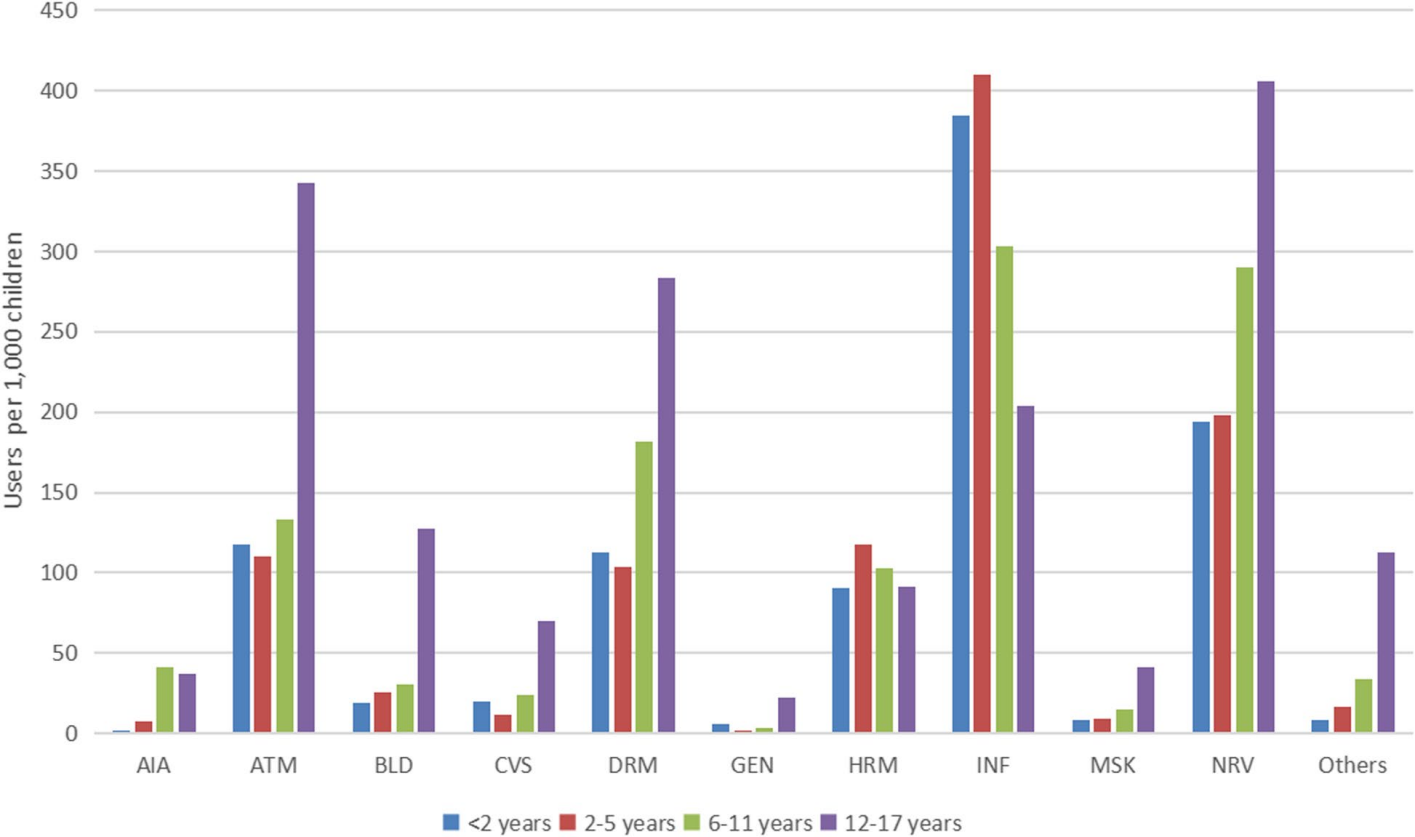
Prevalence of medicine use according to age group. AIA: antineoplastic and immunomodulating agents, ATM: alimentary tract and metabolism, BLD: blood and blood-forming organs, CI: confidence interval, CVS: cardiovascular system, DRM: dermatologicals, GEN: genitourinary system and sex hormones, HRM: systemic hormonal preparations excluding sex hormones and insulins, INF: anti-infectives for systemic use, MSK: musculoskeletal system, NRV: nervous system, RES: respiratory system, SNS: sensory organs, TCM: traditional Chinese medicine

The most widely used medicines were respiratory, anti-infectives, traditional Chinese medicines (TCMs), drugs related to the nervous system, alimentary tract, and dermatological drugs (Fig. 1). Antibiotics accounted for 92.13% (97,925/106425) of all the anti-infectives. However, the prevalence rates for respiratory, TCMs, and anti-infectives were higher for children aged < 6 years than those aged > 6 years. However, the prevalence rates were lower for drugs related to the nervous system, alimentary tract, and dermatological medications for children aged < 6 years.

Table 2 lists all the medicines whose prevalence > 20 users per 1000 person-years in any age group (except for the solvent). As shown in Table 2, the top 3 medications for respiratory were ambroxol hydrochloride, budesonide, and terbutaline. However, the medicine most widely used in children aged < 2 years was ying’er zhike mixture, a hospital preparation used to treat cough. The top 3 anti-infectives were ceftriaxone, cefixime, and cefaclor. The top TCMs were dehydroandrographolide succinate, xiao’er jinqiao granule, and feilike mixture.

**Table 2:** Most commonly used medicines according to age group and prevalence of medicine use per 1000 person-years for 2019–2020

medication	ATC-code	Age group (years)								All children		
		< 2 years		2–5 years		6–11 years		12–17 years				
		2019	2020	2019	2020	2019	2020	2019	2020	2019	2020	Rate ratio (95% CI)^a^
**AIA**		**1.15**	**3.32**	**5.38**	**12.77**	**26.1**	**67.84**	**34.62**	**40.33**	**16.32**	**35.32**	2.164(2.069–2.264)
leuprorelin	L02AE02	0.03	0	0.12	0.27	9.48	36.13	1.94	4.5	3.21	12.18	3.793(3.460–4.159)
**ATM**		**126.75**	**103.38**	**114.58**	**112.46**	**138.94**	**160.35**	**370.08**	**356.8**	**171.96**	**188.7**	1.097(1.081–1.114)
glycyrrhizin	*	5.71	6.04	10.92	11.71	27.65	36.61	46.43	50.26	21.71	28.44	1.310(1.253–1.369)
lansoprazole	A02BC03	0.09	0	0.07	0.03	0.76	0.37	25.87	33.98	5.15	8.87	1.723(1.582–1.876)
pantoprazole	A02BC02	0.09	0	0.08	0.03	2.75	0.51	24.62	11.02	5.5	3	0.545(0.483–0.614)
polyene phosphatidylcholine	*	0.03	0	0.07	0.14	1.56	0.7	18.59	25.94	4	6.93	1.734(1.574–1.910)
vitamin B6	A11HA02	21.37	14.47	15.4	9.08	12.45	5.82	11	5.29	14.66	7.81	0.533(0.495–0.573)
vitamin C	A11GA01	29.56	19.38	21.85	12.72	17.09	7.88	12.07	5.59	19.84	10.27	0.518(0.486–0.552)
**BLD**		**20.22**	**16.32**	**21.24**	**33.47**	**31.4**	**29.99**	**133.5**	**119.94**	**45.26**	**52.48**	1.159(1.124–1.196)
alanyl glutamine	B05XB02	0	0	0.04	0	1.33	0.51	23.77	27.36	4.89	7.2	1.471(1.343–1.612)
mecobalamin	B03BA05	1.24	3.92	2.69	21.85	5.57	10.3	15.25	15.62	5.67	14.45	2.546(2.364–2.742)
**CVS**		**22.64**	**14.86**	**11.66**	**12.14**	**25.75**	**21.05**	**73.82**	**64.85**	**29.32**	**28.76**	0.981(0.941–1.022)
**DRM**		**101.4**	**132.04**	**91.97**	**115.79**	**160.27**	**180.64**	**230.11**	**301.05**	**139.72**	**185.2**	1.326(1.304–1.347)
calamine lotion	*	28.53	33.91	19.06	20.54	13.39	13.51	2.93	3.04	15.87	15.62	0.984(0.930–1.042)
fusidic acid	D06AX01	0.51	5.84	0.51	4.24	0.94	6.22	5.44	32.56	1.57	12.33	7.862(6.968–8.870)
selenium sulfide	D01AE13	0.06	0.66	0.04	0.93	0.27	5.74	0.83	22.56	0.26	7.92	30.581(23.189–40.330)
tacrolimus	D11AH01	2.21	3.38	6.53	10.75	14.04	22.62	16.6	22.04	9.95	16.3	1.639(1.541–1.744)
**GEN**		**6.29**	**4.71**	**1.81**	**1.89**	**3.73**	**3.71**	**24.31**	**19.88**	**7.35**	**7.44**	1.012(0.932–1.100)
**HRM**		**100.31**	**68.21**	**132.17**	**88.99**	**116.74**	**78.36**	**94.59**	**87.05**	**115.37**	**82.61**	0.716(0.700–0.732) ^b^
dexamethasone	H02AB02	84.5	55.08	115.48	68.13	81.81	38.52	34.41	25.51	85.23	46.52	0.546(0.530–0.562)
prednisone	H02AB15	5.17	5.77	7.07	10.2	12.17	16.01	20.2	23.13	10.74	14.72	1.370(1.287–1.459)
**INF**		**407.43**	**334.62**	**441.96**	**347.49**	**349.05**	**225.77**	**218.64**	**185.25**	**366.82**	**267.22**	0.728(0.720–0.737) ^b^
azithromycin	J01FA10	34.42	27.94	66.9	43.16	52.01	23.92	9.68	7.08	46.45	26.09	0.562(0.540–0.585)
cefaclor	J01DC04	114.51	115.59	68.86	55.44	18.03	15.28	10.1	1.85	50.15	37.21	0.742(0.717–0.768)
cefazolin	J01DB04	16.74	26.94	16.82	25.84	12.91	13.87	9.97	6.85	14.36	17.47	1.217(1.150–1.287)
cefdinir	J01DD15	6.62	7.1	13.94	21.74	12.58	18.57	6.71	7.58	10.99	15.26	1.389(1.305–1.477)
cefixime	J01DD08	11.61	11.15	52.31	47.31	83.82	62.94	36.74	36.63	52.08	44.65	0.857(0.830–0.886)
cefmetazole	J01DC09	11.54	36.63	16.45	51.31	11.12	33.96	1.27	7.58	11.22	32.91	2.934(2.788–3.088)
ceftriaxone	J01DD04	92.78	68.75	81.88	68.37	45.86	27.04	17.81	11.12	60.92	41.17	0.676(0.645–0.698)
cefuroxime	J01DC02	44.91	0	71.45	0	58.76	0	13.39	0	52.45	0	
immunoglobulins	J06BA02	30.59	15.53	7.84	3.5	4.94	1.27	3.81	1.49	9.9	3.85	0.389(0.351–0.430)
minocycline	J01AA08	0	0.13	0.01	0.08	0.15	0.28	18.38	21.94	3.52	5.78	1.641(1.478–1.822)
**MSK**		**1.90**	**23.22**	**3.64**	**21.47**	**12.06**	**19.86**	**32.16**	**52.38**	**11.23**	**29.17**	**2.598(2.465–2.738)-**54)
ibuprofen	M01AE01	1.42	22.43	2.85	19.28	8.25	9.88	5.64	2.02	4.74	12.39	2.614(2.411–2.835)
**NRV**		**194.33**	**288.77**	**197.95**	**232.41**	**289.84**	**335.31**	**405.66**	**420.76**	**263.68**	**319.28**	**1.211(1.198–1.224)**
aripiprazole	N05AX12	0.06	0	1.39	2.57	22.49	33.57	19.06	19.98	10.73	16.11	1.501(1.412–1.595)
chloral hydrate	N05CC01	69.45	114.73	40.93	58.17	10.46	13.79	3.06	2.71	29.4	37.72	1.283(1.235–1.333)
clonazepam	N03AE01	8.16	0	20.99	0	39.48	0	20.64	0	24.3	0	
idebenone	N06BX13	5.29	6.04	11.33	13.37	18.28	27.83	45.36	59.79	18.83	28.76	1.527(1.459–1.599)
levetiracetam	N03AX14	0.27	9.42	4.94	17.39	25.66	31.96	17.32	20.81	12.63	21.66	1.715(1.625–1.811)
nalmefene	N07BB05	0	0	0.19	0.03	2.01	0.62	22.53	17.6	4.91	4.73	0.962(0.868–1.067)
nitrazepam	N05CD03	0	31.65	0	11.65	0	10.16	0	2.68	0	11.46	
olanzapine	N05AH03	0.06	0.07	0.06	0.11	0.55	0.76	23.46	12.77	4.62	3.56	0.771(0.687–0.865)
oxcarbazepine	N03AF02	12.51	22.63	15.14	25.46	22.33	33.76	19.42	24.78	17.64	27.44	1.555(1.484–1.630)
paracetamol	N02BE01	50.53	46.05	32.34	15.59	15.2	4.36	7.9	0.43	25.61	12.2	0.476(0.449–0.505)
sertraline	N06AB06	0.03	0	0.07	0.05	1.62	2.59	57.59	48.44	11.38	13.27	1.166(1.094–1.243)
valproic acid	N03AG01	10.34	15.99	23.15	35.72	40.71	52.95	31.36	31.37	27.8	37.28	1.341(1.290–1.394)
**RES**		**522.09**	**428.77**	**454.32**	**454.78**	**326.24**	**241.87**	**201.94**	**139.72**	**379.86**	**305.88**	0.805(0.797–0.814)^b^
ambroxol	R05CB06	110.98	64.37	98.83	63.45	50.29	22.43	9.48	8.93	69.6	37.11	0.533(0.516–0.551)
budesonide	R03BA02	105.72	85.2	81.75	80.48	48.65	58.11	30.16	36.26	66.12	62.93	0.952(0.926–0.978)
cineole with limonene and α-pinene	*	0.12	0.13	0.81	0.33	15.58	12.92	30.92	24.05	10.74	10.22	0.952(0.888–1.021)
desloratadine	R06AX27	0.18	0.93	0.73	3.86	0.85	14.38	1.84	20.48	0.88	10.95	12.373(10.592–14.454)
loratadine	R06AX13	19.34	31.39	35.43	68.84	26.39	28.33	9.5	8.27	25.26	36.18	1.432(1.376–1.490)
mometasone	R03BA07	3.66	3.12	40.5	48.84	47.88	35.12	24.91	11.32	33.77	29.16	0.863(0.829–0.899)
montelukast	R03DC03	15.5	10.68	46.47	54.07	29.39	20.99	12.95	8.73	30.09	26.81	0.891(0.854–0.930)
terbutaline	R03AC03	86.83	64.37	68.32	67.61	29.77	24.93	4.69	2.61	47.93	37.54	0.783(0.756–0.811)
ying’er zhike mixture	*	143.35	140.81	31.62	33.97	3.13	2.87	0.57	0.46	35.42	29.64	0.837(0.804–0.871)
**SNS**		**42.61**	**54.54**	**40.38**	**73.92**	**51.1**	**127.29**	**48.52**	**109.28**	**45.44**	**96.7**	2.128(2.072–2.185)
**TCM**		**329.12**	**241.66**	**381.31**	**279.61**	**291.81**	**187.37**	**250.38**	**234.35**	**321.75**	**235.16**	0.731(0.722–0.740) ^b^
dehydroandrographolide succinate	*	150.27	84.14	163.7	93.29	107.3	43.73	16.6	11.05	117.11	55.94	0.478(0.465–0.490)
feilike mixture	*	13.6	17.65	51.17	53.41	37.59	26.25	5.36	4.53	32.44	28.02	0.864(0.829–0.900)
lanqin oral solution	*	11.58	0	19.77	0	20.39	0	7.48	0	16.3	0	
tanshinone capsule	*	0.03	0	0.1	0.22	1.38	2.34	22.14	33.88	4.62	9.5	2.054(1.884–2.239)
xiao’er chaigui tuire granule	*	54.4	54.21	32.4	21.8	15.1	7.15	1.55	1.36	25.03	16.26	0.650(0.617–0.684)
Xiao’er feire kechuan oral solution	*	12.51	0	25.24	0	10.21	0	0.83	0	14.14	0	
xiao’er qingqiao granule	*	41.77	45.99	46.41	61.34	29.5	32.13	3.03	5.06	32.48	36.04	1.110(1.068–1.152)
**Others**		**5.92**	**14**	**12.86**	**24.34**	**26.1**	**46.99**	**108.7**	**118.08**	**33.73**	**54**	1.601(1.548–1.655)

^a^ Prevalence rate ratio of medications for children in 2020 compared with 2019

^b^ p < 0.001

* Not registered in ATC 2021

Table 2 also shows the changes in the prevalence of medicine use for all children between 2020 and 2019. There were significant decreases in the use of respiratory, anti-infectives, TCMs, and systemic hormones. The prevalence rate of dehydroandrographolide succinate, ambroxol, and dexamethasone, the top 3 medications most widely used in 2019, dropped about 50%, and the total prevalence rate of the major antibiotics dropped nearly 25%. This contrasted with a significant increase in the drugs related to the nervous system and dermatological medicines.

### Inappropriate types of prescriptions

Of the 322,000 prescriptions analyzed, 65,974 were considered inappropriate, reaching a prevalence rate of 20.49%. Among these inappropriate prescriptions, a total of 73,358 inappropriate cases were found, and 6373 prescriptions had more than one case. As shown in Table 3, the top 3 common inappropriate categories included indication-related off-label drug use(67.93%), improper administration frequency (17.80%), and overdosing (11.06%).

**Table 3:** Composition ratio of inappropriate categories

inappropriate categories	overdosing	improper administration frequency	improper administration routes	contraindication	improper diluent volume	medication not allowed for pediatric patients	improper drug combination	repeated administration	medication not match the diagnosis
Composition ratio	11.06%	17.80%	0.71%	0.05%	0.79%	1.11%	0.08%	0.46%	67.93%

As shown in Table 4, inappropriate prescriptions were more in 2019 than in 2020, and the rate was higher (21.71%, 44,421/204606) than in 2020 (18.36%, 21,553/117394) (RR 0.846; 95%CI 0.833–0.858; p < 0.001). In age groups, overdosing, improper administration frequency, improper administration routes, improper diluent volume, and indication-related off-label drug use were more likely seen in patients aged 2–5 years. While contraindications, medicines not allowed for pediatric patients, improper drug combination, and repeated administration were more likely seen in patients aged > 12 years.

**Table 4:** Composition of each inappropriate category

	overdosing	improper administration frequency	improper administration routes	contraindication	improper diluent volume	medication not allowed for pediatric patients	improper drug combination	repeated administration	indication-related off-label drug use	total
year										
2019	75.68%	63.97%	46.36%	97.22%	91.67%	74.54%	62.90%	64.60%	67.10%	67.62%
2020	24.32%	36.03%	53.64%	2.78%	8.33%	25.46%	37.10%	35.40%	32.90%	32.38%
Age groups, years										
< 2	9.97%	18.14%	15.13%	0.00%	10.42%	3.55%	0.00%	2.65%	15.31%	14.98%
2–5	57.72%	33.52%	38.12%	0.00%	46.53%	8.20%	1.61%	12.68%	31.96%	34.85%
6–11	26.91%	22.48%	24.90%	25.00%	35.94%	22.77%	32.26%	32.15%	20.96%	22.12%
12–17	5.39%	25.86%	21.84%	75.00%	7.12%	65.48%	66.13%	52.51%	31.77%	28.06%
categories by the ATC drug classification										
AIA	0.23%	1.21%	5.94%	0.00%	0.00%	6.24%	72.58%	4.42%	0.81%	0.98%
ATM	0.22%	1.98%	2.11%	63.89%	0.17%	10.65%	1.61%	0.00%	12.24%	8.86%
BLD	0.06%	0.88%	8.62%	0.00%	1.04%	0.00%	8.06%	1.77%	6.05%	4.36%
CVS	0.47%	3.58%	0.38%	0.00%	0.00%	0.37%	4.84%	1.47%	3.98%	3.41%
DRM	0.00%	0.57%	0.57%	22.22%	0.00%	0.00%	0.00%	0.29%	7.00%	4.87%
GEN	0.05%	0.08%	0.77%	0.00%	0.00%	16.40%	0.00%	0.00%	0.17%	0.33%
HRM	1.32%	0.13%	11.69%	0.00%	0.00%	0.00%	0.00%	8.55%	0.86%	0.88%
INF	5.35%	8.96%	9.77%	0.00%	92.53%	20.69%	11.29%	0.59%	16.34%	14.33%
MSK	0.09%	0.24%	0.00%	5.56%	0.00%	18.97%	0.00%	2.06%	0.85%	0.85%
NRV	5.03%	15.95%	0.19%	0.00%	0.00%	24.97%	0.00%	5.60%	6.13%	7.87%
RES	78.36%	34.45%	49.04%	0.00%	0.35%	0.12%	1.61%	74.93%	36.29%	40.15%
SNS	0.00%	0.68%	9.20%	8.33%	0.00%	0.00%	0.00%	0.29%	2.81%	2.10%
TCM	8.77%	31.24%	0.96%	0.00%	5.90%	0.49%	0.00%	0.00%	5.92%	10.61%
Others	0.04%	0.05%	0.77%	0.00%	0.00%	1.10%	0.00%	0.00%	0.54%	0.40%

Among all the inappropriate drug use, respiratory medicines accounted for 40.15%, followed by anti-infective drugs (14.33%) and TCMs (10.61%). Contraindication was more likely to appear in the alimentary tract and metabolism drugs. Medication not allowed for pediatric patients was more likely to appear in nervous system drugs, and improper drug combinations were more likely to occur in antineoplastic and immunomodulating drugs.

## Discussion

This paper provides an overview of outpatient prescriptions and inappropriate medicinal use in the Chinese pediatric population. It assesses changes in prescription rates and prevalence of inappropriate medicinal use between 2019 and 2020. The study also identifies significant changes in the use of medicines before and after the outbreak of novel coronavirus pneumonia (COVID-19).

As an effect of COVID-19, the number of outpatient visits in Chinese general hospitals had decreased significantly [17]. For example, taking the author’s hospital, the number of pediatric outpatients in 2020 was just 70.46% of that in 2019. To avoid gatherings and infection, pediatric patients tried to minimize the number of hospital visits, and doctors reduced the use of injected formulations. For the same purpose, doctors were more likely to prescribe more extended courses of medication to patients with chronic diseases to reduce the number of visits, so the spending on drugs per visit increased by 25.14% in this study.

Respiratory diseases are the most common disease in pediatrics [18–20]. This study found that 42.87% (138,054/322000) of children who were prescribed medicines were diagnosed with respiratory diseases. Respiratory medicines, anti-infectives, and TCMs were most commonly used for respiratory diseases in children,and the number and prevalence rate of inappropriate use were also the highest. So the children who have respiratory diseases may have higher risks of inappropriate prescriptions, and doctors should be more cautious when prescribing drugs to these children.

The respiratory medicines were most widely used, and they appeared to be most inappropriately used. Budesonide was associated with severe off-label use related to indication, which accounted for 19.91% of all the improper medication use that related to indication.69.82% (14,604/20916) budesonide was used either for pneumonia, tracheobronchitis, or upper respiratory trace infection. However, no evidence can be present from current research,that inhaled corticosteroids are effective in the treatment of cough due to acute tracheobronchitis and upper respiratory tract infection. Thus, it was not recommended to be used for routine respiratory tract infections [20, 21]. Ambroxol was the second commonly used respiratory medicine, and the majority of ambroxol used was in injection form. In contrast, the use of oral preparations began just after March 2020 but was used only for 21 children all over the year. Ambroxol hydrochloride injection was used 18,598 times in 2019 and 2020, and the prevalence reduced by 46.68% in 2020 than in 2019, with the decrease in the use of all injected formulations. In all the ambroxol hydrochloride injections used, 27.54%(5122/18598) were identified to have been overdosed. The overdosing of ambroxol hydrochloride injection accounted for 63.15% (5122/8111) of overdosing use of all the medicines. Several multi-center surveys on ambroxol hydrochloride injection in China indicated that the off-label drug use, especial off-label administration route, and off-label dosage were common; the incidence was 40–48% and 36–45%, respectively [22, 23]. In the author’s hospital, the off-label administration route of ambroxol was infrequent, and overdosing was lesser than in the hospitals above. Great attention needs to be paid, and more prospective trials are required to identify the effectiveness, safety, and necessity of off-label drug use of respiratory medicines. We believe that if the appropriate use of respiratory medications is increased, the rate of inappropriate prescriptions would be significantly reduced.

Since 2012, China has formally implemented a series of decrees for the clinical use of antibacterial agents [24]. However, the inappropriate use of antibiotics for respiratory tract conditions and viral infectious diseases still existed in pediatric outpatients. Third-generation cephalosporins and second-generation cephalosporins were the most commonly prescribed antibiotic classes, which accounted for 72.80%(71,283/97925) of all antibiotics used. However, these third-generation cephalosporins and second-generation cephalosporins were all WHO watch-group antibiotics and were not indicated to be prescribed for mild respiratory tract infections and viral infections [25]. The prevalence of improper administration frequency, overdosing, and improper diluent volume for injection in antibiotics was lower than 1% due to the strict antibiotic supervision by hospital management. It can be seen that the use of antibiotics under supervision has fetched some results, but more attention should be paid to the selection of antibiotics.

TCMs were widely used in children. Long-term clinical practice has confirmed that TCMs are safe and effective in treating acute upper respiratory tract infections in children [26]. In the case of COVID-19, the advantages of TCMs in treating respiratory diseases have been proved again [27, 28]. In this study, the top 7 TCMs were all used for respiratory infections, in which three were pediatric drugs, whose dosage form and specifications were suitable for pediatric use. The other four TCMs were not pediatric drugs and had no specified doses for children. Doctors could only determine the dosage or administration frequency for these medicines according to their experience. This was also the main problem in the application of TCMs in children.

Dexamethasone was widely used in children, and 16.31% (22,510/138054) pediatric outpatients with respiratory diseases such as pneumonia, tracheobronchitis, or upper respiratory tract infections, were prescribed dexamethasone injection. The glucocorticoids were not recommended for routine use of respiratory diseases in various treatment guidelines in China. They can only be used to relieve symptoms when the patient has wheezing, breathing difficulties, and is at risk of suffocation [18, 29]. Due to the limited information provided by the prescription, it was difficult to retrospectively and accurately determine whether each patient had the indication for the use of dexamethasone. Still, from the high use rate of dexamethasone, it could be inferred that a large number of dexamethasone was used inappropriately. Since the outbreak of COVID-19, the Department of Pharmacy and the Medical Service at the author’s hospital organized a series of training on pneumonia and other respiratory diseases, including the rational use of glucocorticoids in respiratory diseases. The utilization of dexamethasone was reduced by 45.42% in 2020 compared to 2019 in this study, which could indicate that the use of glucocorticoids became more rationally in pediatric outpatients. However, the inappropriate use of glucocorticoids still exists, which requires more attention.

This study observed that only for 1.1% (3628/322000) pediatric outpatients, the cost of drug was covered by basic medical insurance, reflecting that the proportion of medical expenses paid by medical insurance for pediatric outpatients is very low. China has established a basic medical insurance system, covering more than 95% of the population in 2020. However, at present, the basic medical insurance for children mainly focuses on inpatient and serious illness, but most children who fall ill occur in the outpatient department. On the other hand, the medicines out of the medical insurance catalog need to be paid on their own. Commercial health insurance for Children in China is still at the initial stage, and the coverage rate is very low [2, 30, 31]. So it was suggested that optimizing the design of basic medical insurance policies and formulating more favorable policies for children is quite necessary.

## Conclusion

In general, compared to 2019, inappropriate drug use in pediatric outpatients in 2020 had significantly improved in hospital but is still common, especially in off-label use related to indication, doge, and administration frequency. In response to these situations, the Department of Pharmacy and the Medical Service at the author’s hospital has adopted a series of interventions, such as specialized training for doctors, essential drug monitoring, and a list of off-label drug use, etc. In this process, the clinical rational drug use monitoring system plays a vital role in data monitoring, analysis, inappropriate prescription interception, etc. Since 2021, the clinical rational drug use monitoring system has started to intercept the inappropriate prescriptions of essential monitoring of drugs, which will significantly improve children’s drug use’s rationality, safety, and effectiveness.

### Limitations

This study only involved one tertiary academic hospital, a teaching hospital, that can reflect the highest medical level in this province. Although it was strongly suggested by experts in China to establish the regional prescription circulation network between medical institutions and uniform prescription review and evaluation, there is still no successful case nationwide. Therefore, there is no multi-center data and a homogeneous prescription evaluation system among medical institutions currently.

The hospital did not implement electronic medical records before 2021, and doctors could only write medical records on paper. If the patient did not purchase the medicine prescribed by the doctor in the hospital, the information of the prescription could not be retained and could not be involved in this study.

## Data Availability

Not applicable.
